# Hybrid Approach for Improving the Performance of Data Reliability in Cloud Storage Management

**DOI:** 10.3390/s22165966

**Published:** 2022-08-10

**Authors:** Ali Alzahrani, Tahir Alyas, Khalid Alissa, Qaiser Abbas, Yazed Alsaawy, Nadia Tabassum

**Affiliations:** 1 Faculty of Computer and Information Systems, Islamic University Madinah, Madinah 42351, Saudi Arabia; 2 Department of Computer Science, Lahore Garrison University, Lahore 54000, Pakistan; 3 Networks and Communications Department, College of Computer Science and Information Technology (CCSIT), Imam Abdulrahman Bin Faisal University (IAU), P.O. Box 1982, Dammam 31441, Saudi Arabia; 4 Department of Computer Science and Information Technology, Virtual University of Pakistan, Lahore 54000, Pakistan

**Keywords:** cloud computing, cloud storage, reliability, performance, secure data management, modeling

## Abstract

The digital transformation disrupts the various professional domains in different ways, though one aspect is common: the unified platform known as cloud computing. Corporate solutions, IoT systems, analytics, business intelligence, and numerous tools, solutions and systems use cloud computing as a global platform. The migrations to the cloud are increasing, causing it to face new challenges and complexities. One of the essential segments is related to data storage. Data storage on the cloud is neither simplistic nor conventional; rather, it is becoming more and more complex due to the versatility and volume of data. The inspiration of this research is based on the development of a framework that can provide a comprehensive solution for cloud computing storage in terms of replication, and instead of using formal recovery channels, erasure coding has been proposed for this framework, which in the past proved itself as a trustworthy mechanism for the job. The proposed framework provides a hybrid approach to combine the benefits of replication and erasure coding to attain the optimal solution for storage, specifically focused on reliability and recovery. Learning and training mechanisms were developed to provide dynamic structure building in the future and test the data model. RAID architecture is used to formulate different configurations for the experiments. RAID-1 to RAID-6 are divided into two groups, with RAID-1 to 4 in the first group while RAID-5 and 6 are in the second group, further categorized based on FTT, parity, failure range and capacity. Reliability and recovery are evaluated on the rest of the data on the server side, and for the data in transit at the virtual level. The overall results show the significant impact of the proposed hybrid framework on cloud storage performance. RAID-6c at the server side came out as the best configuration for optimal performance. The mirroring for replication using RAID-6 and erasure coding for recovery work in complete coherence provide good results for the current framework while highlighting the interesting and challenging paths for future research

## 1. Introduction

Cloud computing is the delivery of different services through the Internet. These resources include tools and applications like data storage, servers, databases, networking, and software. These features are the requirements and concerns raised by a larger audience on the internet. The world wide web has carved new paths and approaches to utilize the concept of the digital world at an optimum level. Cloud computing has emerged with this conception and has fulfilled many expectations. It has also brought new questions and challenges for this new ecosystem. Cloud computing serves corporate needs and caters to individual users, making its adaptation swifter than expected [1]. The private and public cloud segregation is practical enough to provide services at a limited exclusive level or in a large and non-exclusive manner.

Similarly, the service model presented by cloud computing very justly provides core services to individual and corporate groups. Additional value-added services are as per the user’s choice but are not a compulsion. The core services [2], i.e., infrastructure as a service (IaaS), platform as a service (PaaS) and software as a service (SaaS) are providing the essential facilities, including the provision of suitable infrastructure (computing, storage, network), an operating platform to utilize the infrastructure and software to perform certain tasks. This configuration is applicable to an individual and a large corporate entity. Therefore, as it is a highly distributed ecosystem, the pricing strategy is also cost-effective and based on the pay-as-you-use phenomenon. The essential requirements at a cost effective and logical pricing model play a key role in spreading the cloud userbase. International technology firms started cloud services that led to quality and performance-driven competition and resulted in consumers’ benefiting [3].

Microsoft Azure, Google Cloud Platform, Amazon EC2, Oracle, IBM, Cisco and many more cloud service providers (CSP) are bringing new services, pricing models and facilities to the consumer [4].

As discussed, cloud platforms are a service-focused computing environment with distributed shared resources and uses when you need a service model [5]. Considering the service model structure, the applications designed for cloud platforms are divided into multi-tier architectures. These tiers are designed on the respective service model, i.e., IaaS, PaaS and SaaS. However, due to the rapid growth in services and to competitive advantages among cloud service providers (CSPs), the emerging services are not able to cater to 3-tier architecture; therefore, multi-tier architecture, or in simple words N-tier architecture for *n* number of services, is the suitable candidate for application development design. Provisioning multiple services in different tiers or in one single tier generates the vital importance of sustainable performance [6].

The multi-tier model is a robust solution for providing an integrated service pack in a single machine to the end-user. Microsoft Azure and Amazon cloud services are based on the multi-tiered cloud environment. The benefit of the multi-tier cloud model is an optimal workload capacity and multi-node management, i.e., multiple users are able to be managed with different service requests in one multi-tiered model [7]. To ensure the operationalization and continuity of the services, a virtualization mechanism is being used in each tier to provide more stable operation with low energy consumption and minimal storage requirements. Virtualization is applicable in single tier or can be implemented in a complete multi-tiered environment depending on the usage and application of the target services. These services acquire a stable operating structure consisting of communication, data processing, security and storage. The balancing of these factors is called data reliability [8].

To ensure the maximum benefits and stable operations in a data centric cloud environment, it is essential to address the data storage, processing and navigation issues in the utmost detail to provide multi-dimensional solutions and frameworks. One core segment of this exercise is the reliability modeling for the cloud environment that leads to the development of the basis for cloud mining, predictive analytics, decision support systems, intelligent configuration systems and cloud ecosystem management. Assuming that all factors have a high ranking except the data reliability, then all other factors are compromised, and the purpose of the whole operation results in a data failure [9]. All other factors are secondary while considering data reliability, and the dependencies on this specific factor are precise and huge; therefore, it is almost essential to develop relevant frameworks and mechanisms to enhance the reliability score in cloud storage environments [10].

Moreover, it also contains the recoveries from failure, backups and fault tolerance capabilities to ensure smooth operations. In cloud computing, these servers may have different physical locations and resources. They are connected through routers and internet by using virtualization, and the physical differences in locations are insignificant. Multiple servers can be treated as one giant server for the user in a virtual environment consisting of collective resources for computing, storage and the network. A hard disk drive (HDD) can be categorized into two types, i.e., HDD that can have volume storage, speed and connectivity. The second type of storage is a solid-state disk (SSD) which brings more speed, storage capacity and security. These types are having an impact on the software applications running the performance and response times. Currently a series of SSDs or a mix of SSDs and HDDs are used in data centers. The series are known as RAID for high performance and robust data processing, including concurrent virtualization and data influxes from different resources [11].

As mentioned earlier, beyond free services, the public cloud offers a versatile and flexible pricing model engaging the concept of “pay as you use”, making it possible to build resources according to capacity and demand. This pricing model leads to a flexible cloud platform that is highly scalable as per the growing demand and technology requirements, along with a facility requiring minimal capital expenditure. Moreover, all service providers provide end-user data protection and recovery capabilities, which include redundancy to prevent losses in the event of any catastrophe. On the other hand, keeping a backup system is an expensive and technical exercise which is not suitable to every end user. A server with good computing and network capabilities always needs a fast, flexible and stable physical storage mechanism [12].

**Figure 1:**
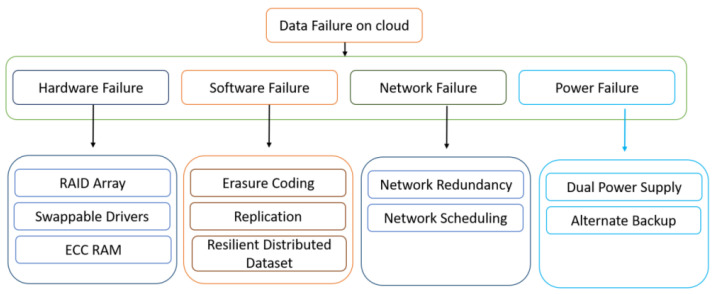
Data Failure in Cloud computing.

There are different types of data failures in cloud computing such as software failure, hardware failure, power failure and network failure. All of these factors can lead to data failure and can affect cloud service failures. In a conventional file system, the failure may be a network outage, machine failure, drive failure and data corruption, as shown in Figure 1.

Erasure coding is mainly used for protecting the data from failures in large-scale storage. It is also used to detect and correct errors in cloud computing. In erasure codes, a file can be divided into equal chunks. It also added the parity chunks that can be restored to recover the original file. Erasure codes can be divided into two categories: Maximum Distance Separable (MDS) and non-MDS [13].

The replication technique focuses more on the cloud computing reliability process to maximize data availability and reliability. Low latency and minimal latency can be reached by consuming bandwidth overcapacity on the network. The lost data need to be restored in the alternative storage medium by retaining the emphasis on reliability. Furthermore, restoration is reactively and proactively divided into two groups. For replication, there are two techniques used. The replica will be generated with the reactive method after the failure. In a constructive method, the replica will be generated before failure occurs. In Static Replication, the total number and location of replicas are fixed. Random replication is used in HDFS, GFS, RAM Cloud and Windows Azure [14]. In dynamic replication, replicas are generated and removed dynamically. The management, position, and deletion of replica productions are autonomous processes that rely on user requirements to improve usability, durability, cost, bandwidth, latency, energy, storage efficiency and execution time.

The storage segment of cloud computing is based on the data centers in terms of physical infrastructure. By a definition from Google, a data center is a cluster of buildings with various functions, numerous server machines and communication equipment to be linked together to develop a common environment with common maintenance and security needs. In terms of components, the physical structure can be divided into three main categories, i.e., server machines, storage and reliability. The server machines are designed for heavy processing, communication and storage facilities [15]. The multi-function, multi-user and multi-tiered physical structure is the most vital and common segment to be looked at for reliability and storage optimization in a cloud environment.

## 2. Problem Statement

Data storage in cloud environments is a big challenge for data reliability and trust issues. Both key attributes play a critical role in the survival and growth of cloud services and in attaining the trust level of cloud users. The tendency of engaging cloud storage services is expanding continuously, and the data management in cloud environments is becoming a big challenge in terms of data reliability, security and accessibility. This research aims to formalize a hybrid approach for data reliability in cloud storage management and also introduces a conceptual framework to improve reliability storage efficiency and latency in accessing data over cloud computing.

## 3. Research Motivation

This research has formulated the following research motivations;

- Reliability of data requires optimizing durability and data availability.
- Durability mitigates permanent failures and mitigates temporary failures by availability.
- In cloud data centers, different methods are used to increase the fault tolerance of the storage system.

## 4. Significance of Our Study

Replication is a proven process in cloud computing and data centers to maintain data reliability and efficiency. Mirroring and erasure coding both are valid methods and have their respective advantages and disadvantages. This research provides a hybrid framework to engage in mirroring and erasure coding for replication based on the advantages and performance of each method. Therefore, the proposed framework provides the optimal data replication and respective features to deal with the heavy volumes and associated risks.

## 5. Research Objectives

The following objectives are defined for this research;

- Designing cloud storage reliability assurance to evaluate storage properties.
- To produce an autonomous storage management model for improving storage efficiency.
- Formulating a model for data reliability in cloud storage management.

## 6. Literature Review

Cloud storage systems are composed of large numbers of hardware and software components. Failures are the norm rather than the exception in cloud storage systems. Any failures such as hardware failures, power outages, software glitches, maintenance shutdowns or network failures in the cloud storage system will raise temporary data unavailability events and sometimes lead to permanent data loss. In spite of these failures, to provide reliable service to the customers, various fault-tolerant mechanisms are employed. To meet the large-scale storage needs of clients, cloud defines virtual storage using Network Attached Storage (NAS) and Storage Area Network (SAN) [16].

The networked storage NAS and SAN are easily scalable in terms of both performance and capacity, and hence are highly influential in cloud storage systems. They use a distributed file system to organize data for storage and provide controlled data access to clients. Distributed File System (DFS) spreads data in a storage cluster which is composed of thousands of nodes. DFS applies data redundancy to improve the fault tolerance of cloud storage systems, and it spreads redundant data into nodes from different failure zones [17]. DFS is also designed to ensure the durability, availability and I/O performance of the storage according to the client’s Service Level Agreement (SLA).

Any failures in cloud storage systems mentioned above may lead to unavailability events from time to time. Whenever an unavailability event occurs, it activates data recovery to maintain durability and data availability. The data redundancy mechanisms employed in cloud storage systems are replication and erasure coding. Replication maintains multiple copies of data on distinct nodes from different failure domains. Replication is a straightforward fault-tolerant method. However, it is not an efficient solution for big data due to the volume of data. Erasure coding is a storage-efficient alternative reliability method [18].

Strauss has mentioned the importance of reliability based on the availability of data, which is becoming a critical factor when considering or measuring the performance of any cloud service; therefore, it is becoming essential to consider the reliability methodologies while developing the cloud’s structure [19]. Zhang has mentioned the importance of virtual machines, i.e., the configuration of virtual machines in a manner that supplements the reliability and availability of the data. It also has an impact on the performance of the cloud services, and therefore, the configuration of virtual machines is becoming more and more critical [20]. The role of infrastructure as a service in terms of the storage and performance of the cloud services is discussed by Vishwanath and Nagappan. They have presented reliability metrics for the performance evaluation of the cloud services focused on storage and data [20]. In continuation, Bauer has highlighted the critical parameters for the quality of service. The reliability metrics presented by various scholars are applied on certainly quality parameters to evaluate the impact of various variables on the reliability and availability of the data [21].

The reliability and recovery of the data has not been evaluated at the infrastructure level only, and various scholars have developed and tested different algorithms to address various critical challenges. In the same year, Cheng proposed another framework for the reduction in failure and dependencies by improving the system’s reliability. The basis of this framework is also rooted in the infrastructural services in cloud computing [22]. This framework has been used to evaluate the performance of cloud services in an independent mode and develop rankings for the same.

The research related to reliability and availability has extended into the prevention of failures, identification of failures, performance prediction and defensive methodologies. Sharma has highlighted the importance of such indirect factors on the overall performance and reliability of the data. The preventive measures and predictive maintenance of the computing system have a long-term impact on the strategies for cloud computing storage, replication and recovery methodologies [23].

The topic of cloud computing storage has become a more comprehensive area for researchers as it incorporates hardware or infrastructure as a service (IaaS), virtualization and computation capabilities, and more importantly, the configuration of virtual machines. Nachiappan has discussed the virtual machines scenarios of multiple configurations and the relevant impact on the performance of the cloud services. The importance of this work is related to the challenges of big data in cloud computing, and specifically related to the storage reliability and availability in case of virtual machine failures or weak configurations. They also discussed the role of security, preventive measures and storage methodologies such as static replication, dynamic replication, mirroring and erasure coding, to be specific [24].

The Popular Hadoop Distributed File System (HDFS) uses three replicas. Hence, it can tolerate any two simultaneous failures with a storage overhead of 3x. The most popular Reed–Solomon can manage any four simultaneous failures with 1.4x storage overhead. Even though the storage efficiency of erasure coding sounds appealing, data recovery/repair in erasure coding involves enormous resource consumption. For example, data recovery in Reed–Solomon code increases disk I/O and network bandwidth by 10x compared to replication (increased resource consumption due to data recovery also impacts read performance) [25]. Data recovery in replication has a limited impact on resource consumption and read performance. Data recovery issues of erasure coding prevent it from being more pervasive in cloud storage systems. For example, in a 3000-node production cluster of Facebook, erasure coding can replace replication for only 8% of data. In the case that 50% of the data are replaced with erasure code, the repair network traffic will saturate cluster network links [26].

When there is a failure in cloud storage systems, the objects that are resided in the failed zone will enter into a degraded mode. A delay is applied to recover any degraded objects to avoid any unnecessary repair. Degraded objects will remain in degraded mode from the time of failure utill complete recovery. Any data read request to a degraded object in replication is handled by redirecting requests to the next available replica. On the other hand, in erasure coding, a degraded object is reconstructed on the fly. In replication, the object is recovered by copying it from the next available replica, whereas in erasure coding, the object is recovered using the data reconstruction of any other k available chunks [27].

## 7. Solution Design and Implementation

### 7.1. Conceptual Description of the Solution

The purpose of this framework is to develop provisions for the cloud users who are dealing with a heavy volume of data/big data in a distributed environment, as managing multiple virtual machines can engage both successful techniques instead of selecting one specific one. The framework is designed in three main layers to cater to the objectives described in the previous sections. It is also important to note that different erasure coding and data replication algorithms are already available with proven results and performance; therefore, the scope of this research work is to build a working environment in which various algorithms can work as per the requirement. Moreover, new and emerging algorithms are also manageable [28].

In cloud computing, the main challenge is managing data prolifically and securely. As the cloud computing platform is designed for a distributed and shareable environment, at all service levels, i.e., IaaS, PaaS and SaaS, the concepts of distribution, load management and latency control are the main pillars. At the infrastructure level, the storage of data is completed using various methods, and the most prominent and effective methods are data replication and erasure coding. Both the methods are focused on data reliability, availability and profiling to ensure the data’s integrity and security. Both methods have pros and cons related to the swift recovery of data, latency and the mechanism through which both methods store the data. This research proposes a hybrid framework that uses both methods, i.e., replication and erasure coding, to provide optimum data integrity [29].

The expected outcome is fewer data losses, failures and swift recoveries using optimum methodologies. Erasure coding uses RAID 5/6, while replication uses mirroring on RAID-1. Replication using mirroring may use more space but provides better results in terms of the reliability of data due to the redundant mechanism, while erasure coding is more economical on storage and provides swift recovery in case of failure. The fault tolerance and recovery capacity of erasure coding is better than replication, but on the other hand, replication provides more reliability and integrity though at a slower pace, with an expense in terms of storage by using more space.

Most manufacturers now include a system restoration disc computer to help reduce the difficulties and frustrations of fixing a system malfunction. This instance is manageable by configuring Kubernetes in a cloud configuration, and the proposed framework uses CNI (cloud network interface) and CSI (cloud storage interface) to identify the healthy nodes and perform an assessment based on the virtual machine configuration, i.e., the compute, storage and network properties available with the configuration manager. In other words, the default configuration of all virtual machines resides with the configuration manager, while the dynamic status of the virtual machines is captured through CSI and CNI interfaces that transform into the health status of the virtual machines, i.e., in the case that, due to a recovery instance, erasure coding is activated on virtual machine number 3, the dynamic value of its storage will increase. The CSI and CNI interfaces pass the value to the VM health controller, and in the case of any room being made available for dynamic replication or erasure coding by creating an instance on another virtual machine, it will start the load balancing accordingly to create space for the current instance.

**Figure 2:**
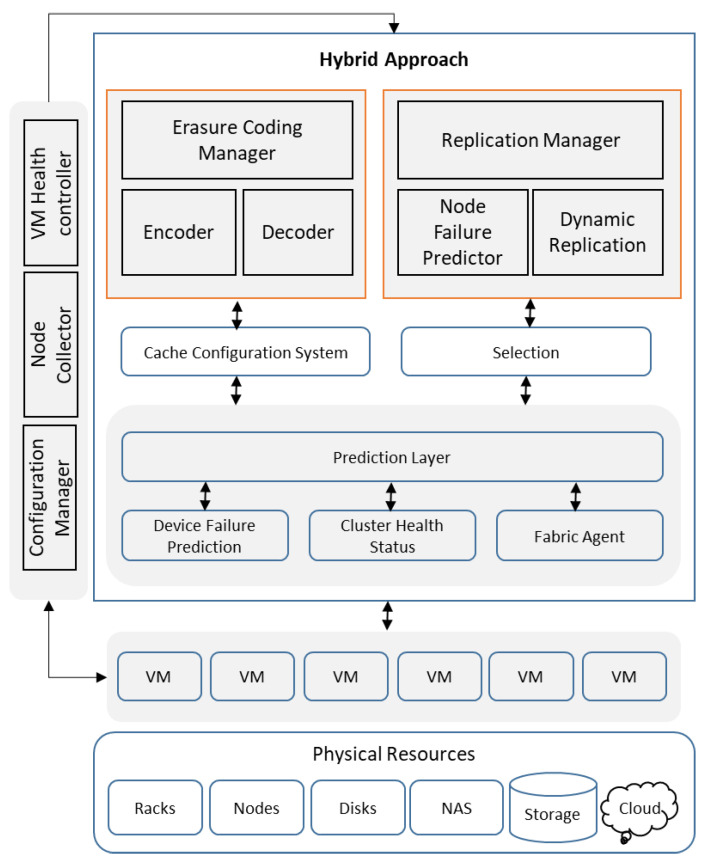
Conceptual description of the proposed solution.

In the case that all VMs are busy and not able to accommodate the current instance, then instead of crashing the system, the current instance will be in the cache cue. Therefore, as soon as the virtual machine is available in processing terms, i.e., suitable compute, storage and network properties, then it will start the recovery or replication procedure as planned. The physical resource layer consists of hardware variants in terms of storage devices, i.e., racks, nodes, disks, etc. As we are considering the cloud platform, therefore, this layer physically resides with the cloud service provider in the case of a public, community or hybrid cloud. In the case of a private cloud or a corporate data center, this layer shall be categorized as in-prim hardware services. It was mentioned before that erasure coding works on RAID 5/6 and replication uses RAID-1, and the physical resource layer manages both. The accessibility to this layer is provided through virtualization in terms of different virtual machines. The cloud platform is flexible enough to provide a customized configuration of each virtual machine, though the overall capacity of all virtual machines is dependent on the physical resource available. To select between two storage services, i.e., erasure coding and replication, this layer is formulated to provide data cluster health and device failure possibilities using a fabric agent for host management. It is notable here that device failure predictions are based on the possible failure of the erasure coding or replication method. To elaborate further, if replication uses HDFS for mirroring and faces a hardware, bandwidth or logical fault, the fabric agent will provide a health alert for the current data cluster and evaluate the possibility of failure to predict the event. The same process is applicable for erasure coding, e.g., in the case that erasure coding is using hyper-convergent storage for video streaming and facing high latency, hardware failure or a split modulation problem, the fabric agent will perform the same function as mentioned before. The objective of this module is to take necessary action, i.e., switching to the other method within a minimal timeframe without disturbing the data operations and management, as shown in Figure 2.

This layer is linked with the prediction layer and operates in accordance with the fabric agent’ instructions. This framework takes erasure coding and replication management modules as hosts, while the fabric agent is the host controller to assign tasks and operations received from the virtual machines or cloud users. As depicted in the diagram, the hybrid layer is split into main modules, i.e., erasure coding management and replication management, with two connecting functions: a cache configuration system and selection for the prediction layer. This layer also contains the respective algorithms for erasure coding as well as for replication. New algorithms can be added into the same modules, and therefore, the optimum solution for storage management is possible with the most recent and best algorithms.

The management sub-module contains the algorithms with the encoder and decoder sub-modules. This framework works on this assumption that the available algorithms are proven and tested; therefore, the encoder and decoder are able to perform their respective functions. Virtual erasure coding, HDFS erasure, hyper-convergent erasure and Reed Solomon erasure are a few examples. From the prediction layer, the fabric agent will provide the task to the host, i.e., erasure coding management with the data cluster health status, and by default, both methods will be used based on the capacity and availability of physical resources. In the case of replication management failure, the erasure coding management module will resume the task and start recovering data from the replication module. For this purpose, the replication module has one dedicated node in the erasure coding module. In the case of a bad health flag on the data cluster or the predictive failure of a device linked with the replication management module, the erasure coding module will start recovering and assuming data tasks through the dedicated node.

Replication management is connected with the prediction layer through the selection function for two main tasks. The first is to take the backup of a data cluster with a bad health status declared by the fabric agent. This data cluster is used by erasure coding or replication management module, and this module shall complete the mirroring of that cluster with both methods. It is also possible that certain parts or segments of the cluster need to be backed up. Using various algorithms, e.g., genetic algorithms, multi-tier data algorithms, mirroring algorithms, etc., will ensure a timely backup. The second task is related to the failure of erasure coding. The replication module will take the backup of the encoded data from the erasure coding management. A dedicated node shall also be available in the replication management module to ensure data cluster health status replication, predictive device failure, and possible erasure coding failure or high latency. This switching process is seamless and quick to provide an uninterrupted data service without failure. While in the case of the failure of any of the two components, a swift and timely recovery is possible through this hybrid approach.

### 7.2. Design of The Solution

The system monitoring layer maintains the stable working of the whole structure by evaluating the changing configurations, node adjustments and virtual machines’ status as per the configuration manager. In case of a particular node failure, the load is distributed by engaging close with virtual machines to provide an instance for the recovery process to streamline the system again.

### 7.3. Validation Prototype

We have used the Streamlit (version 3.10 for python created by Pablo Fonseca for github, San Francisco, CA, USA) data streaming and erasure coding. Streamlit is a GNU-based algorithm that is primarily designed to develop erasure coding for high-volume distributed data management. It uses machine learning to engage statistical methods in neural networks to select a suitable encoding algorithm for the data cluster. The validation of the proposed framework has been completed by using a video streaming scenario, as it is heavy in volume and also requires a swift and smooth recovery without interruption.

As per the scope of this research, a hybrid approach is designed to formulate a balance between replication and erasure coding to gain optimum storage reliability and recovery performance. In the validation segment, purpose failures were induced to evaluate the performance and activation of erasure coding to provide a swift recovery and continuation of service. Replication and erasure coding are tested with heavy-volume data processing as in normal scenarios. The cloud storage methodology with replication works fine and provide successful results. The aim of this research, as explained in the previous section, is to provide such a hybrid method that can generate robust performance in case of high data volumes and have the capacity for failure tolerance and the continuation of operations without compromising the storage reliability and recovery characteristics.

The scenario is developed using the existing technology and best practices under consideration. Current cloud storage methodologies extensively use RAID technology for the physical storage and manipulation of the data. Different RAID levels have proven to have performance and reliability pros and cons. To evaluate the proposed framework, this research has engaged RAID-0 to RAID-6 for data diversification and spreading while the most commonly used RAID-5 and RAID-6 are used for the replication and erasure coding. RAID-5 and RAID-6 both are configured for the mirroring purpose, while the erasure coding is deployed on RAID-6 only for focused and more interpretable results. Further grouping between RAID-1 and RAID-5/6 is categorized on the basis of failure tolerance. RAID-1 is grouped with RAID-5/6 with a fault tolerance of 1, with 200 gb and 133 gb of capacity, respectively. The other group contains RAID-1 and RAID-5/6 with a fault tolerance of 2, with a capacity of 300 gb and 150 gb, respectively. Dataset, RAID configuration, RAID grouping, and mirroring and erasure coding with various capacities have been performed. For operations, the data are used with various protocols that provide the more complex scenarios at the administrative level to ensure the robustness and impact of the proposed framework for reliability and recovery using replication and erasure coding.

### 7.4. Validation Structure

The dataset we have used is the KDD12 dataset for the purpose of validation, consisting of approximately 1,074,992 records. A random selection of 322,98 records has been used for the mirroring and erasure coding experiment on various RAIDs, as depicted in the following table. The RAIDs for the replication experiment are configured as shown in Table 1.

For replication, the following blocks are configured on RAID-5 and RAID-6 as shown in Table 2. The mentioned RAIDs contain a cluster of 53,480 records on each configuration for replication.

For mirroring and erasure coding, the following configuration is applied on the RAID-6, as shown in Table 3.

The final configuration for the scenario is given in Table 4. Notably, the scope of this research is limited to reliability and recovery propositions, but there are many other parameters which are not evaluated in this scope. Therefore, the mirroring cycles, backup and functional RAID classification are not defined.

As is visible, RAID-5 and RAID-6 reciprocate RAID-1 mirroring, while RAID-3 and RAID-4 use mirroring, but in a block and striping format. If RAID-1 (mirroring without striping) will impact erasure coding, then upgrading the other RAID formats becomes easy and more logical. Therefore, from a data spread point of view, all RAIDS are engaged, while the configuration above is being used from a reliability and recovery perspective. The failure limit is also defined for each RAID classification, and with the exception of RAID-0 and RAID-6, all other RAIDS have a failure threshold on one drive, while RAID-6 is configured as a failure threshold on two drives. There is a parity diversification for each RAID as well, ranging from no parity, shared parity, dedicated parity, distributed parity and double distributed parity.

The parity range also significantly impacts the replication and performance of the RAID; therefore, it is highly important to evaluate various configurations to understand the validation of the proposed framework. Double distributed parity is assigned to RAID-6, while RAID-5 uses distributed parity, but both are complex parity cases. Therefore, with the other configurations mentioned in the previous section, the scenarios developing at RAID-5 and RAID-6 are challenging and depict the real-life complexity level and robustness requirements at an optimum level.

In cloud computing, there are other storage methods, but as mentioned earlier, in the literature, we have identified the best practices for storage, and RAID is one of the most usable methodologies in various cloud configurations.

Initially, data parameters are used for training purposes to engage intelligence, which is required to develop autonomy as the system obtains maturity. The switching between replicated mirroring and erasure coding is essentially based on the intelligent flagging and understating of the anomaly patterns by the machine itself.

Therefore, the parameters are trained to learn the anomaly flags, and these learning datasets are generated through a number of iterations. To make the learning more independent, the column names are also dynamic, and the machine learns the columns and transforms a training structure every time instead of fixing on specific columns, meaning that the same learning is applicable on multiple cloud and RAID configurations without any modifications, as it is going to pick the columns dynamically to formulate the learning parameters.

## 8. Performance Evaluation of the System

As per the scenario explained in the previous section, the initial rationale is linked with the data’s framing and evaluation. Data are linked with various protocols assuming that various communication mechanisms are attached to the cloud, and therefore, the nature and source of data needs to be rationalized before proceeding towards the learning and testing of the system for mirroring and recovery. The following are the data framing results over multiple iterative engagements to evaluate various possibilities, as shown in Table 5. The results show the normalization of data to process for the pre-training adjustments. The results show the tcp and udp source using ftp and http protocols. though the private channel does not reach the normal value. Further data processing and learning shows the private data channel is generating rejection of tcp instead of proceeding towards restoration, as shown in Table 6. The other examples show that the ftp data are again reaching a normal value but not proceeding towards mirroring or restoring. Similarly, another case has generated an association pattern for mirroring and showing restoration of tcp. 

### 8.1. Training Set Results

**Figure 3:**
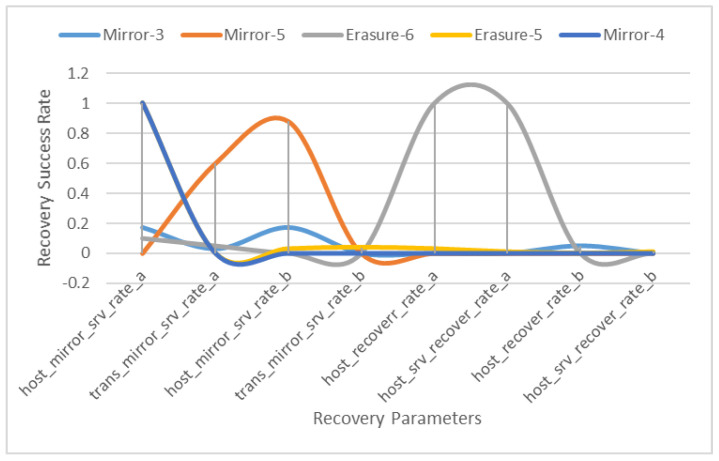
Training Results.

The training dataset has generated the categories and columns shown in Table 5. The dataset is segregated into the host mirror’s server rate and the transition server rate, while the recovery is also split into these types, i.e., host recovery rate and host server recovery rate. The purpose is to manage the server-side storage and the VM-level storage. Another aspect covered in this activity is the mirroring not only required for data at rest for servers but which is also applicable to data in transient. The results provide the values for the proceedings. The following graph summarizes the results as shown in Figure 3. The host mirror’s server rate for mirror-5 configuration shows an overall promising result, and on the other hand, the recovery using the erasure-6 configuration is also prominent.

Data are linked with various protocols assuming that various communication mechanisms are attached with he cloud, and therefore, the nature and source of the data needs to be rationalized before proceeding towards the learning and testing of the system for mirroring and recovery. The following are the data framing results over multiple iterative engagements to evaluate various possibilities. The results show the normalization of the data to be processed for the pre-training adjustments. The results show that the tcp and udp source using ftp and http protocols though the private channel does not reach the normal value.

Further data processing and learning shows the private data channel generates rejection of tcp instead of proceeding towards restoring. The other examples show the ftp data again reaching a normal value but not proceeding towards mirroring or restoring. Similarly, another case has generated an association pattern for mirroring and showing the status of the restoration of tcp.

The training dataset has generated the categories and columns shown in the aforementioned table. The dataset is segregated into the host mirror’s server rate and the transition server rate, while the recovery is also split into these types, i.e., host recovery rate and host server recovery rate. The purpose is to manage the server-side storage as well as the VM-level storage. Another aspect covered in this activity is mirroring, which not only required for data at rest for servers but for data in transient. The results provide the values for the proceedings. The following graph summarizes the results. The host mirror’s server rate for the mirror-5 configuration shows an overall promising result, but on the other hand, the recovery using the erasure-6 configuration is also prominent.

The further processing of the data focuses on mirroring and erasure coding with reference to the mirror, transit and restoration. These parameters are evaluated with the same modes, i.e., host mirroring, transit mirroring, host recovery and host recovery at the server end. The results are showing lower rejection in the case of mirroring, while rejection is showing higher rates in the b recovery configuration at the server and virtual machine level. Similarly, erasure coding and mirroring show significant trends in host mirroring at the server end, while the restoration is higher on the recovery modes. The erasure and mirroring show significant results at the server end, while these results are not as good when considering data in transit.

Now, for the scenario built on different configurations of the RAID, the results show the analysis of RAID-1 to RAID-6 with respect to erasure coding, erasure coding with parity and mirroring combinations with all parameters. RAID 1 to 4 are used in the default configurations, while RAID-5 and RAID-6 are used in different combinations/configurations, i.e., RAID-5a and RAID-5b, while RAID-6 is configured in three different modes labelled as a, b and c. The mirroring instance engages all the configurations. As is visible in the graph, mirroring RAID-6c and RAID-6a are on the top for mirror and replication. Both RAID modes are hybrid in nature as configured previously. The instance occurring as ER1—erasure coding instance—engages RAID-6c, RAID-6b and RAID-6a as the most significant configurations, while RAID-4 also has a prominent value that shows that the best performance of erasure coding is in hybrid mode along with the mirroring instance, but in the case of only erasure coding, the RAID-4 value shows the impactful behavior of erasure coding for recovery and reliability. The second instance, or erasure coding ER2, takes RAID-6, RAID-6b and RAID-5b as the parameters, working in a hybrid mode only. This proves that the proposed framework provides significant results regarding reliability and swift recovery performance in cloud storage.

The results show the best performance and coherence in a hybrid manner, i.e., mirroring and then handing over to erasure coding for recovery. The Erasure-6 and Mirror-5 configurations are hybrid modes. Data at rest and data in transit are both addressed successfully.

### 8.2. Mirror and Erasure Coding Results

Further processing of the data focuses on mirroring and erasure coding with reference to the mirror, transit and restoration, as shown in Table 7. These parameters are evaluated with the same modes, i.e., host mirroring, transit mirroring, host recovery and host recovery at the server end.

**Figure 4:**
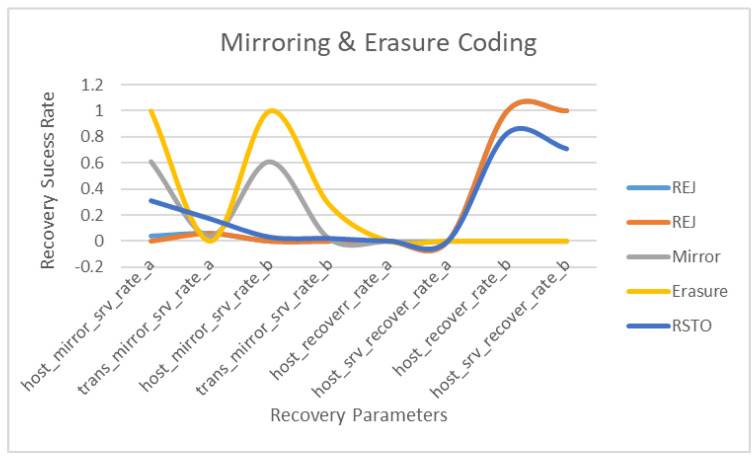
Mirroring and Erasure Coding Graph on RAID.

The results in Figure 4 show lower rejection in the case of mirroring, while rejection shows a higher rate in the b recovery configuration at the server and virtual machine level. Similarly, erasure coding and mirroring show significant trends in host mirroring at the server end, while the restoration is higher on the recovery modes. The erasure and mirroring show significant results at the server end, while these results are not as good while considering data in transit.

**Figure 5:**
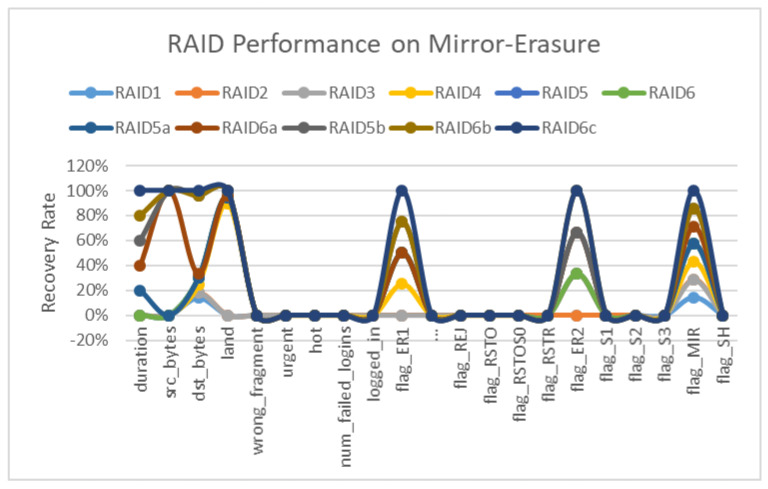
Mirroring and Erasure Coding Graph.

The second instance of erasure coding ER2 takes RAID-6, RAID-6b and RAID-5b as the parameters, which only work in a hybrid mode, as shown in Figure 4. This proves that the proposed framework provides significant results regarding reliability and swift recovery performance in cloud storage. After the training and testing, the dataset’s structure shows a minimal error rate, with erasure coding having value ranges between zero and one, as shown in Table 8. Figure 5 shows other parameters along with erasure flag having a value of one in four out of five values. The one in which erasure does not show results also has a source volume of zero. Therefore, erasure does not perform any operations with zero volume. While in the other four cases, the data volume is tangible, this also proves the application of erasure coding with volume, and the same is applicable for mirroring, as the real power and purpose of mirroring is exposed with volumes, as shown in Table 9. 

### 8.3. Erasure Coding Results

**Figure 6:**
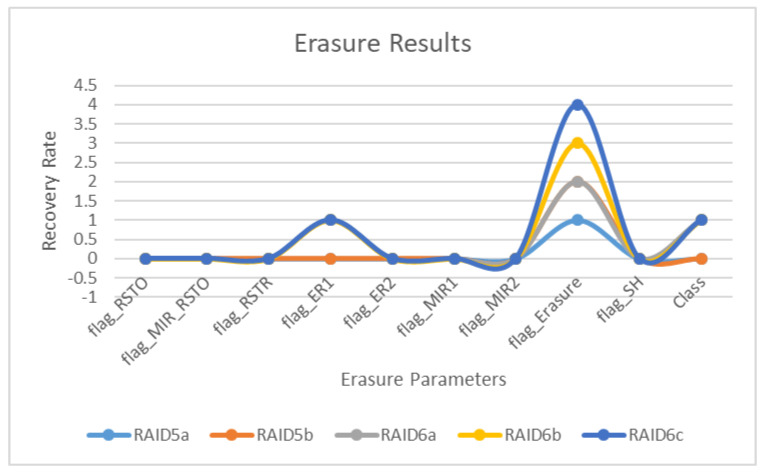
Erasure coding results.

Now, for the scenario built on different configurations of the RAID, results are shown in Table 10 for the analysis of RAID-1 to RAID-6 concerning erasure coding, erasure coding with parity and mirroring combinations with all parameters, as shown in Figure 6. The mirroring instance engages all the configurations. RAID 1 to 4 are used in the default configurations, while RAID-5 and RAID-6 are used in different combinations/configurations, i.e., RAID-5a and RAID-5b, while RAID-6 is configured in three different modes labeled as a, b and c. The graph shows that RAID-6c and RAID-6a are on the top for mirroring and replication in mirroring. Both RAID modes are of a hybrid in nature as configured previously. The instance occurring as erasure coding instance ER1 is engaging RAID-6c, RAID-6b and RAID-6a as the most significant configurations, while RAID-4 also has a prominent value that shows the best performance of erasure coding is in the hybrid mode along with the mirroring instance, but in the case of only erasure coding, the RAID-4 value shows the impactful behavior of erasure coding for recovery and reliability. 

### 8.4. Training Results

The focused results for the erasure coding as shown in Table 11 are extracted with RAID configurations, as is visible in the table above, and the same is also more visible in the graph below. The ER instances are visible on all levels in the case of RAID-6c, while all other RAIDs do not show a significant performance on any parameter. Therefore, it is right to say that the most successful hybrid configuration for replication and erasure coding is RAID-6c, the second best can be RAID-6b, followed by RAID-6a and RAID-5a. RAID-6c has a significant value and is more comprehensive as it responds to all other parameters, even if those parameters have lower or nominal values, but at least it depicts the rationale between different parameters. The base class developing in this scenario also reaches the value of one, which also reciprocates the significant value of flag_erasure. The overall results prove this research’s hypotheses regarding the reliability and recovery impact of a hybrid modulation between replication and erasure coding.

### 8.5. System Summary

In cloud computing, various service providers extend different productivity tools to enhance the user experience. The proposed framework evaluates the need for dynamic replication using mirroring or the recovery process using an erasure coding module. The replication and relevant tasks can be configured to have a better visual response by managing CNI and CSI, as mentioned before. The ongoing activity is viewable on virtual machines.

Both processes will consume computation and storage utilization; therefore, in virtualization monitoring, the visible processes of Reco (Recovery) are visible along with Opm (Optimization) and local. Virtual machines engaged in replication shall switch to local, and after replication, shall have the status of optimized, and in the case of recovery activity using erasure coding, the status will change to recovery.

The structure of the dataset after the training and testing shows a minimal error rate, with erasure coding having value ranges between zero and one. Table 10 shows other parameters along with erasure flag having a value of one for four out of five values. The one in which erasure is not showing a result also has a source volume of zero; therefore, erasure does not perform any operations with zero volume. While in the other four cases, the data volume is tangible, this also proves the application of erasure coding with volume, and the same is applicable for mirroring, as the real power and purpose of mirroring is exposed with volumes.

The focused results for the erasure coding are extracted with RAID configurations, as is visible in Table 11 and moreso in the graph below. The ER instances are visible on all levels in the case of RAID-6c, while all other RAIDs do not show a significant performance on any parameter. Therefore, it is right to say that the most successful hybrid configuration for replication and erasure coding is RAID-6c, the second best can be RAID-6b, followed by RAID-6a and RAID-5a. RAID-6c not only has a significant value but also is more comprehensive, as it is responding to all other parameters even if those parameters have lower or insignificant values, but at least it is depicting the rationale between different parameters. The base class developed in this scenario also reaches the value of one, which reciprocates the significant value of flag_erasure. The overall results prove the hypotheses of this research regarding the reliability and recovery impact of a hybrid modulation between replication and erasure coding.

## 9. Conclusions

Cloud computing provides different solutions for data safety, backups and replication. All these methods are independent and work separately, although these methods and tools have common parameters and can work together. The inspiration of this research is based on the development of a framework that can provide a comprehensive solution for cloud computing storage in terms of replication, and instead of using formal recovery channels, erasure coding was proposed for this framework, which in the past proved itself as a trustworthy mechanism for the job. The proposed framework provides a hybrid approach to combine the benefits of replication and erasure coding to attain the optimal solution for storage, specifically focused on reliability and recovery.

The overall results show the significant impact of the proposed hybrid framework on cloud storage performance. RAID-6c at the server came out as the best configuration for optimal performance. The mirroring for replication using RAID-6 and erasure coding for recovery work in complete coherence and provide good results for the current framework, while highlighting the interesting and challenging paths for future research.

## Figures and Tables

**Table 1:** Storage structure.

RAID Type	Description	Min. Drives	Range for Fault Tolerance	Parity
RAID-0	Striping without mirroring	2	None	No Parity
RAID-1	Mirroring without striping	2	01 drive failure	No Parity
RAID-2	Striping with ECC	3	01 drive failure	Shared Parity
RAID-3	Striping (Byte)	3	01 drive failure	Dedicated Parity
RAID-4	Striping (Block)	3	01 drive failure	Dedicated Parity
RAID-5	Striping (Block)	3	01 drive failure	Distributed Parity
RAID-6	Striping (Block)	4	02 drive failures	Double Distributed Parity

**Table 2:** RAID-5 configuration structure.

RAID-5			
A1	B1	C1	P1
B2	C2	P2	A2
C3	P3	A3	B3
P4	A4	B4	C4

**Table 3:** RAID-6 configuration structure.

RAID-6					
A1	B1	C1	D1	P1	Q1
B2	C2	D2	P2	Q2	A2
C3	D3	P3	Q3	A3	B3
D4	P4	Q4	A4	B4	C4

**Table 4:** Storage configuration.

RAID-Configuration	Type	Fault Domains	Failure Tolerate	Data Volume	Required Capacity
RAID1	Mirror	-	1	100 gb	200 gb
RAID 5/6	Erasure Code	4	1	100 gb	133 gb
RAID1	Mirror	-	2	100 gb	300 gb
RAID 5/6	Erasure Code	6	2	100 gb	150 gb

**Table 5:** Data frames results.

Drives	Iterations																		
	Duration	protocol_type	service	Flag	src_bytes	dst_bytes	wrong_fragment	Urgent	num_failed_logins	dst_host_same_srv_rate	dst_host_diff_srv_rate	dst_host_same_src_port_rate	dst_host_srv_diff_host_rate	dst_host_serror_rate	dst_host_srv_serror_rate	dst_host_rerror_rate	dst_host_srv_rerror_rate	Label	difficulty_leve
0	0	tcp	ftp_data	SF	491	0	0	0	0	0.17	0.03	0.17	0	0	0	0.05	0	normal	20
1	0	udp	other	SF	146	0	0	0	0	0	0.6	0.88	0	0	0	0	0	normal	15
2	0	tcp	private	S0	0	0	0	0	0	0.1	0.05	0	0	1	1	0	0	Neptune	19
3	0	tcp	http	SF	232	8153	0	0	0	1	0	0.03	0.04	0.03	0.01	0	0.01	normal	21
4	0	tcp	http	SF	199	420	0	0	0	1	0	0	0	0	0	0	0	normal	21

**Table 6:** Reference adjustment results.

Drives	Iterations																		
	Duration	protocol_type	Service	Flag	src_bytes	dst_bytes	wrong_fragment	urgent	num_failed_logins	num_failed_logins	dst_host_same_srv_rate	dst_host_diff_srv_rate	dst_host_same_src_port_rate	dst_host_srv_diff_host_rate	dst_host_serror_rate	dst_host_srv_serror_rate	dst_host_rerror_rate	label	difficulty_leve
0	0	tcp	private	REJ	0	0	0	0	0	0.04	0.06	0	0	0	0	1	1	Neptune	21
1	0	tcp	private	REJ	0	0	0	0	0	0	0.06	0	0	0	0	1	1	Neptune	21
2	2	Tcp	ftp_data	SF	12983	0	0	0	0	0.61	0.04	0.61	0.02	0	0	0	0	normal	21
3	0	icmp	eco_i	SF	20	0	0	0	0	1	0	1	0.28	0	0	0	0	saint	15
4	1	tcp	telnet	RSTO	0	15	0	0	0	0.31	0.17	0.03	0.02	0	0	0.83	0.71	mscan	11

**Table 7:** Training Set Results.

host_mirror_srv_rate_a.	trans_mirror_srv_rate_a	host_mirror_srv_rate_b	trans_mirror_srv_rate_b	host_recoverr_rate_a	host_srv_recover_rate_a	host_recover_rate_b	host_srv_recover_rate_b	Class	complexity_level
0.17	0.03	0.17	0	0	0	0.05	0	normal	20
0	0.6	0.88	0	0	0	0	0	normal	15
0.1	0.05	0	0	1	1	0	0	neptune	19
1	0	0.03	0.04	0.03	0.01	0	0.01	normal	21
1	0	0	0	0	0	0	0	normal	21

**Table 8:** Mirroring and erasure coding.

host_mirror_srv_rate_a.	trans_mirror_srv_rate_a	host_mirror_srv_rate_b	trans_mirror_srv_rate_b	host_recoverr_rate_a	host_srv_recover_rate_a	host_recover_rate_b	host_srv_recover_rate_b	Class	complexity_level
0.04	0.06	0	0	0	0	1	1	neptune	21
0	0.06	0	0	0	0	1	1	neptune	21
0.61	0.04	0.61	0.02	0	0	0	0	normal	21
1	0	1	0.28	0	0	0	0	ER1	15
0.31	0.17	0.03	0.02	0	0	0.83	0.71	MIR	11

**Table 9:** Erasure coding results.

flag_ER1	…	flag_REJ	flag_RSTO	flag_RSTOS0	flag_RSTR	flag_ER2	flag_S1	flag_S2	flag_S3	flag_MIR	flag_SH
0	…	0	0	0	0	0	0	0	0	1	0
0	…	0	0	0	0	0	0	0	0	1	0
0	…	0	0	0	0	1	0	0	0	0	0
1	…	0	0	0	0	0	0	0	0	1	0
1	…	0	0	0	0	0	0	0	0	1	0
…	…	…	…	…	…	…	…	…	…	…	…
0	…	0	0	0	0	1	0	0	0	0	0
0	…	0	0	0	0	0	0	0	0	1	0
1	…	0	0	0	0	0	0	0	0	1	0
0	…	0	0	0	0	1	0	0	0	0	0
1	…	0	0	0	0	0	0	0	0	1	0

**Table 10:** Erasure Coding after Training Results.

Duration	src_bytes	dst_bytes	land	wrong_fragment	Urgent	Hot	num_failed_logins	logged_in	num_compromised	flag_RSTO	flag_RSTOS0	flag_RSTR	flag_S0	flag_S1	flag_S2	flag_S3	flag_Erasure	flag_SH	Class
0	0	491	0	0	0	0	0	0	0	0	0	0	0	0	0	0	1	0	0
1	0	146	0	0	0	0	0	0	0	0	0	0	0	0	0	0	1	0	0
2	0	0	0	0	0	0	0	0	0	0	0	0	1	0	0	0	0	0	1
3	0	232	8153	0	0	0	0	0	1	0	0	0	0	0	0	0	1	0	0
4	0	199	420	0	0	0	0	0	1	0	0	0	0	0	0	0	1	0	0

**Table 11:** Erasure and replication results.

flag_RSTO	flag_MIR_RSTO	flag_RSTR	flag_ER1	flag_ER2	flag_MIR1	flag_MIR2	flag_Erasure	flag_SH	Class
0	0	0	0	0	0	0	1	0	0
0	0	0	0	0	0	0	1	0	0
0	0	0	1	0	0	0	0	0	1
0	0	0	0	0	0	0	1	0	0
0	0	0	0	0	0	0	1	0	0

## Data Availability

The data used in this paper can be requested from the corresponding author upon request.
